# *Yuxiensis granularis* gen. et sp. nov., a Novel Quellkörper-Bearing Fungal Taxon Added to *Scortechiniaceae* and Inclusion of *Parasympodiellaceae* in *Coronophorales* Based on Phylogenetic Evidence

**DOI:** 10.3390/life11101011

**Published:** 2021-09-25

**Authors:** Digvijayini Bundhun, Dhanushka N. Wanasinghe, Sajeewa S. N. Maharachchikumbura, Darbhe J. Bhat, Shi-Ke Huang, Saisamorn Lumyong, Peter E. Mortimer, Kevin D. Hyde

**Affiliations:** 1 Centre for Mountain Futures (CMF), Kunming Institute of Botany, Honghe County 654400, China; 6371105502@lamduan.mfu.ac.th (D.B.); peter@mail.kib.ac.cn (P.E.M.); 2 Innovative Institute of Plant Health, Zhongkai University of Agriculture and Engineering, Haizhu District, Guangzhou 510225, China; 3 Center of Excellence in Fungal Research, Mae Fah Luang University, Chiang Rai 57100, Thailand; shike.hua13@lamduan.mfu.ac.th; 4 CIFOR-ICRAF China Program, World Agroforestry Centre, Kunming 650201, China; 5 School of Life Science and Technology, University of Electronic Science and Technology of China, Chengdu 611731, China; sajeewa@uestc.edu.cn; 6 No. 128/1-J, Azad Housing Society, Curca, P.O., Goa Velha 403108, India; bhatdj@gmail.com; 7 Research Center of Microbial Diversity and Sustainable Utilization, Chiang Mai University, Chiang Mai 50200, Thailand; saisamorn.l@cmu.ac.th; 8 Department of Biology, Faculty of Science, Chiang Mai University, Chiang Mai 50200, Thailand; 9 Academy of Science, The Royal Society of Thailand, Bangkok 10300, Thailand; 10 CAS Key Laboratory for Plant Biodiversity and Biogeography of East Asia (KLPB), Kunming Institute of Botany, Chinese Academy of Sciences, Kunming 650201, China

**Keywords:** 2 new taxa, 1 new combination, *Hypocreomycetidae*, phylogeny, *Sordariomycetes*, Yunnan

## Abstract

An undetermined saprobic fungal taxon from Yunnan (China) is revealed as a new genus in *Scortechiniaceae* (*Coronophorales*). The novel taxon, *Yuxiensis*, is characterized by immersed to erumpent, semi-globose ascomata, which are not surrounded by any tomentum or conspicuous subiculum, a subcylindrical quellkörper in the centrum, clavate asci with long pedicels and allantoid hyaline ascospores with granular contents. Maximum likelihood and Bayesian posterior probability analyses based on LSU, ITS, *tef1* and *rpb2* sequence data depict a close phylogenetic relationship of the new genus to *Pseudocatenomycopsis*, hence, confirming its placement in *Scortechiniaceae*. *Parasympodiellaceae*, thus far belonging to *Parasympodiellales*, is transferred to *Coronophorales* based on multi-gene phylogenetic evidence. Additionally, the *incertae sedis* monotypic genus *Arthrocristula* is treated as a synonym of *Parasympodiella*, with *Arthrocristula hyphenata* recombined as *Parasympodiella hyphenata* comb. nov., as the type strain of *Arthrocristula hyphenata* clusters inside the *Parasympodiellaceae* clade along with other *Parasympodiella* taxa.

## 1. Introduction

Members of *Coronophorales* are generally found in terrestrial habitats. These fungi occur as saprobes on woody substrates, with no specific host per se, and their diversity spans tropical and temperate regions [1,2,3,4]. The order is accommodated in *Hypocreomycetidae* and it includes six families, viz. *Bertiaceae*, *Ceratostomataceae*, *Chaetosphaerellaceae*, *Coronophoraceae*, *Nitschkiaceae* and *Scortechiniaceae*, classified based on molecular data and morphology [2,5,6,7,8].

*Scortechiniaceae* was introduced by Huhndorf et al. [9] to accommodate three saprobic genera, *Euacanthe*, *Neofracchiaea* and *Scortechinia* using morphological characteristics and LSU sequence data. Mugambi and Huhndorf [2] later added six additional genera, *Biciliospora*, *Coronophorella*, *Cryptosphaerella*, *Scortechiniella*, *Scortechiniellopsis* and *Tympanopsis* to the family. All genera formed a well-supported clade in a multi-gene phylogeny based on LSU, *tef1* and *rpb2* sequence data [9]. Interestingly, all these taxa contain a distinctive phenotypic character in their centrum, namely, the quellkörper, which demarcates them from taxa belonging to other families in the order [2,9]. The quellkörper has been described as a subcylindrical to inverted-conical structure located in the centrum, made up of a mass of thick-walled, hyaline cells tightly arranged in a circular manner [2,9,10]. It is usually attached to the roof of the ascoma, orienting downward, and can extend until the base of the sporocarp [2,9,10]. The structure is mainly thought to be involved in ascospore discharge [2,9,10]. The quellkörper has, over the years, been considered and justified by taxonomists as the primary family-level character [2,3,9].

The monotypic taxon, *Pseudocatenomycopsis,* was then recently added to *Scortechiniaceae* based on LSU and ITS sequence data. The genus is only known in its asexual morph [11]. Latest taxonomic revisions of the family conducted by Huang et al. [12] revealed that *Cryptosphaerella* had to be transferred to *Sordariomycetes* genera *incertae sedis* based on the morphology of its type. The taxa *Neocryptosphaerella* and *Pseudocryptosphaerella* have instead been introduced to accommodate the *Cryptosphaerella* taxa having sequence data in GenBank at present [12].

Another order, *Parasympodiellales*, belonging to the same subclass, *Hypocreomycetidae*, was established by Hernández-Restrepo et al. [13], along with its type family, *Parasympodiellaceae*, to accommodate *Parasympodiella*, whose taxa formed a monophyletic clade in their phylogenetic tree. Members of *Parasympodiellales* are only known in their asexual morphs and are mainly characterized by sympodial conidiogenous cells and thallic-arthric conidia [13,14,15,16]. Furthermore, they usually have a stylaspergillus-like synasexual morph [13,16]. In the multi-gene phylogenetic analyses conducted by Hyde et al. [8], it was observed that *Parasympodiellales* clustered as sister to *Scortechiniaceae* (*Coronophorales*), following which the authors mentioned the status of this order requiring further revision.

Similarly, many species of *Sordariomycetes* still require taxonomic revision, especially those which have so far been identified based on morphology alone. Such is the case for *Arthrocristula hyphenata*, belonging to the monotypic genus *Arthrocristula* [17], currently placed in *Ascomycota* genera *incertae sedis* [18]. DNA sequence data for *A. hyphenata* were recently retrieved by Vu et al. [19] and deposited in GenBank. With both the morphology and molecular data for the taxon now available, it becomes largely feasible to determine the correct placement of *A. hyphenata*.

The present study aims to introduce a new fungal genus, *Yuxiensis*, in *Scortechiniaceae*, collected on a woody host in Yuxi (Yunnan, China) based on morphology and phylogenetic analyses of combined LSU–ITS–*tef1*–*rpb2* sequence data. Furthermore, *Parasympodiellaceae* is included in *Coronophorales* based on phylogenetic evidence. *Arthrocristula* is synonymized under *Parasympodiella*, with *Arthrocristula hyphenata* combined as *Parasympodiella hyphenata* Bundhun & K.D. Hyde, comb. nov., and is accommodated in *Parasympodiellaceae* based on DNA sequence data analyzed in the present study.

## 2. Materials and Methods

### 2.1. Specimen Collection and Morphological Studies

Dead twigs of an undetermined deciduous host were collected from Yuxi, Yunnan Province, China, during the dry season in May 2019. The samples were taken to the mycology laboratory at the Kunming Institute of Botany, in a plastic Ziploc bag and stored inside a paper envelope. External examinations were made using a Motic SMZ 168 Series stereo-microscope. Morphological characters were examined by hand sectioning of sporocarps and placed on water-mounted glass slides. Microscopic photography was conducted using a Nikon ECLIPSE 80i compound microscope (Nikon, Tokyo, Japan) fitted with a Canon EOS 600D camera. The structures which were observed and measured include diameter, height, shape, and color of ascomata; ascomatal wall’s width and cell structure; quellkörper shape; shape, length, and width of asci and shape, size, and ornamentation of ascospores. The Tarosoft (R) Image Frame Work version 0.9.7. program was used for the measurements of photomicrograph structures. Images used for figures were processed with the Adobe Photoshop CS6 Extended version 13.0.1 software (Adobe Systems, San Jose, California). The holotype was deposited in the herbarium of Cryptogams Kunming Institute of Botany Academia Sinica (HKAS). Both Facesoffungi [20] and Index Fungorum [21] numbers were obtained.

### 2.2. DNA Extraction, PCR Amplification and Sequencing

No culture could be obtained for the collected sample despite several trials on various media, including malt extract agar, potato dextrose agar, corn meal agar, or water agar under different incubation conditions. Therefore, DNA was extracted directly from the fruiting bodies of the fungus as outlined by Wanasinghe et al. [22].

According to the manufacturer’s instructions, DNA was extracted from fresh sporocarps using the Biospin Fungus Genomic DNA Extraction Kit-BSC14S1 (BioFlux, P.R. China). Primers LR0R/LR5 [23], ITS5/ITS4 [24], EF1-983F/EF1-2218R [25], and fRPB2-5f/fRPB2-7cR [26,27] were used to amplify the DNA sequences of the partial 28S large subunit rDNA (LSU), internal transcribed spacers (ITS), translation elongation factor 1-α (*tef1*), and RNA polymerase II second largest subunit (*rpb2*). The total volume of 25 μL contained 12.5 μL of 2× PCR Master Mix with dye (0.1 U Taq Polymerase/μL, 500 μm dNTP each), 20 mM Tris-HCl (pH 8.3, 100 mM KCl, 3 mM MgCl_2_), 1 μL of each primer, 9.5 μL of double-distilled water, and 1 μL (100–500 ng) of DNA template.

The PCR protocols were programmed as described in Wanasinghe et al. [28]. The PCR products were verified by staining with ethidium bromide on 1% agarose electrophoresis gels. They were then purified according to the company protocols and DNA sequencing was performed at Shanghai Sangon Biological Engineering Technology & Services Co. (Shanghai, P.R. China). Forward and reverse DNA sequence data were obtained and analyzed. Consensus sequences were generated using the SeqMan software (DNAStar, Inc., Madison, WI, USA). The newly acquired sequence data from the present study were deposited in GenBank for subsequent studies [29] (Table 1).

### 2.3. Phylogenetic Analyses

Verified sequences were initially used for BLASTn analyses, following which closely related sequences were downloaded from GenBank based on BLAST similarities and relevant publications [2,8] (Table 1). Alignment of each locus was performed using MUSCLE in MEGA X (Molecular Evolutionary Genetics Analysis), using default conditions for gap openings and gap extension penalties. It was then improved whenever necessary in the BioEdit v.7.0.5.2 software [30].

Maximum likelihood (ML) and Bayesian posterior probability (BYPP) analyses were conducted using both individual and combined datasets. Prior to ML analysis, the sequence alignments were converted from FASTA into PHYLIP format using the ALTER (alignment transformation environment, http://www.singgroup.org/ALTER/, accessed on 30 August 2021) bioinformatics web tool [31]. They were then used to generate ML trees using RAxML-HPC2 on XSEDE (v.8.2.10) [32] with the GTRGAMMA substitution model and bootstrapping with 1000 replicates.

The BYPP analysis was generated using Markov Chain Monte Carlo sampling in MrBayes v3.1.2 [33,34]. MrModeltest v.2.3 [35] was used to estimate the best evolutionary model for each gene region under the Akaike Information Criterion (AIC) implemented in PAUP v.4.0b10 [36]. The best-fit model was determined as GTR+I+G for LSU, *tef1,* and *rpb2* while GTR+G for ITS. Six simultaneous Markov chains were run for 4.5M generations with trees sampled every 100th generation. The first 20% of generated trees were the burn-in phase and discarded. The remaining 80% of trees were used to calculate posterior probabilities in the majority rule consensus tree. Phylograms were configured in FigTree v.1.4.0 [37] and modified in Microsoft PowerPoint (2013). The final alignments and phylogenetic tree were deposited in TreeBASE, submission ID: 28713 (http://www.treebase.org/ , accessed on 30 August 2021).

## 3. Results

### 3.1. Phylogenetic Analyses

**Figure 1:**
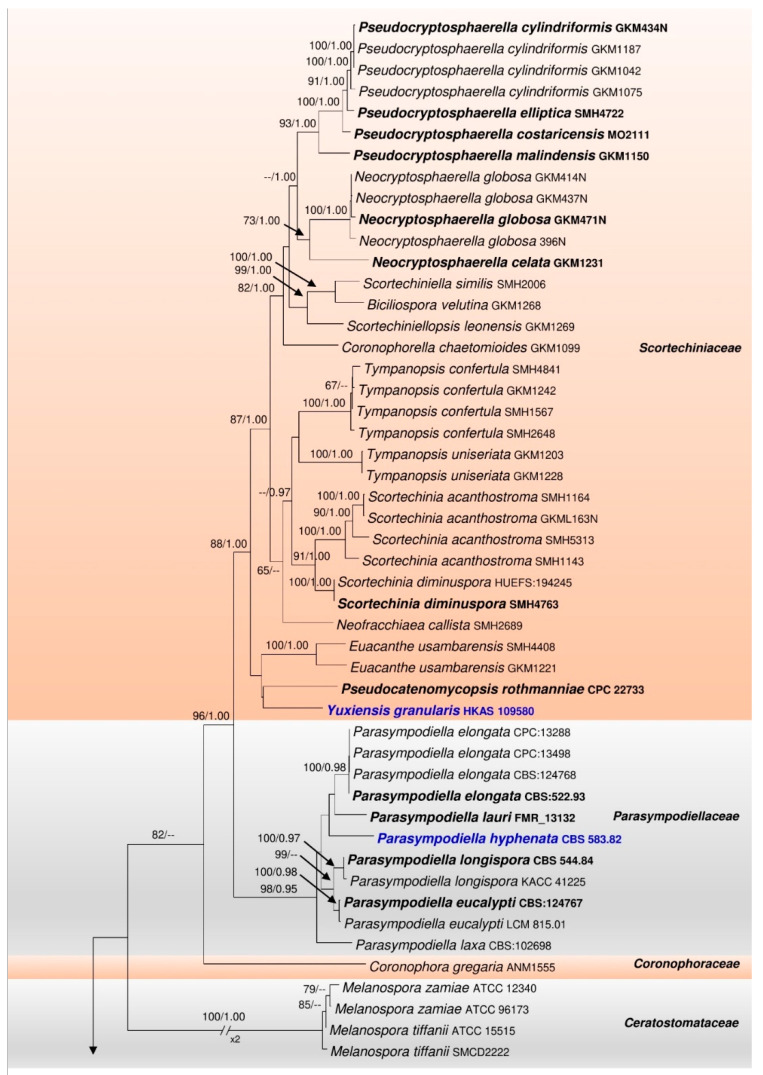

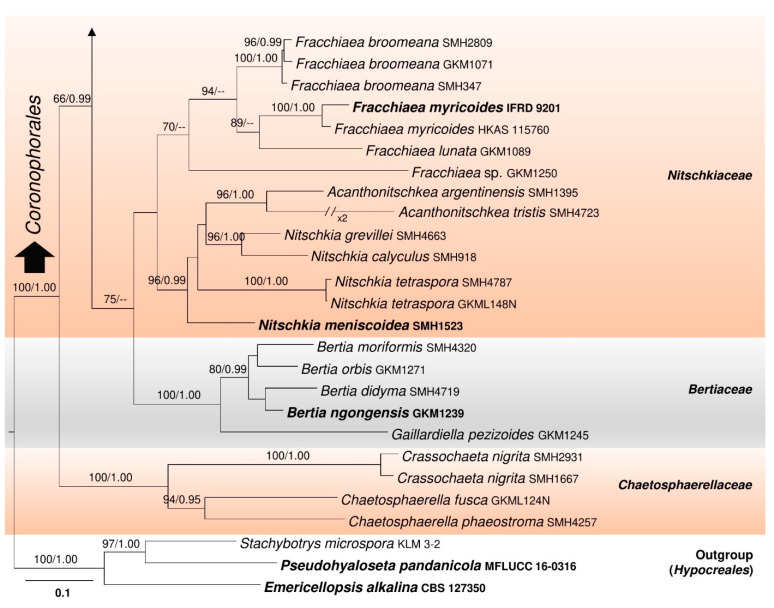
Phylogram generated from maximum likelihood (RAxML) based on LSU–ITS–*tef1*–*rpb2* matrix for *Coronophorales***.** The tree is rooted with *Emericellopsis alkalina* (CBS 127350), *Pseudohyaloseta pandanicola* (MFLUCC 16-0316) and *Stachybotrys microspora* (KLM 3-2). Maximum likelihood bootstrap (≥65) and BYPP (≥0.95) supports are shown, respectively, above or below the branches. Type strains are in bold while novelty and the recombined taxon are in blue.

The final concatenated LSU–ITS–*tef1*–*rpb2* alignment (Figure 1) comprised 75 strains including the outgroup taxa *Emericellopsis alkalina* (CBS 127350), *Pseudohyaloseta pandanicola* (MFLUCC 16-0316), and *Stachybotrys microspora* (KLM 3-2). The manually adjusted dataset consisted of 3845 characters including gaps (LSU: 1086, ITS: 768, *tef1*: 813, *rpb2*: 1178). The best scoring RAxML tree with final optimization had a likelihood value of −41,633.775219. The matrix had 2266 distinct alignment patterns, with 49.47% of gaps and completely undetermined characters. Estimated base frequencies were as follows: A = 0.236728, C = 0.275120, G = 0.286267, T = 0.201885, with substitution rates AC = 1.265283, AG = 3.588580, AT = 1.636499, CG = 1.073052, CT = 7.584875, GT = 1.000000. The gamma distribution shape parameter α = 0.342349 and Tree-length = 7.680873.

**Figure A1:**
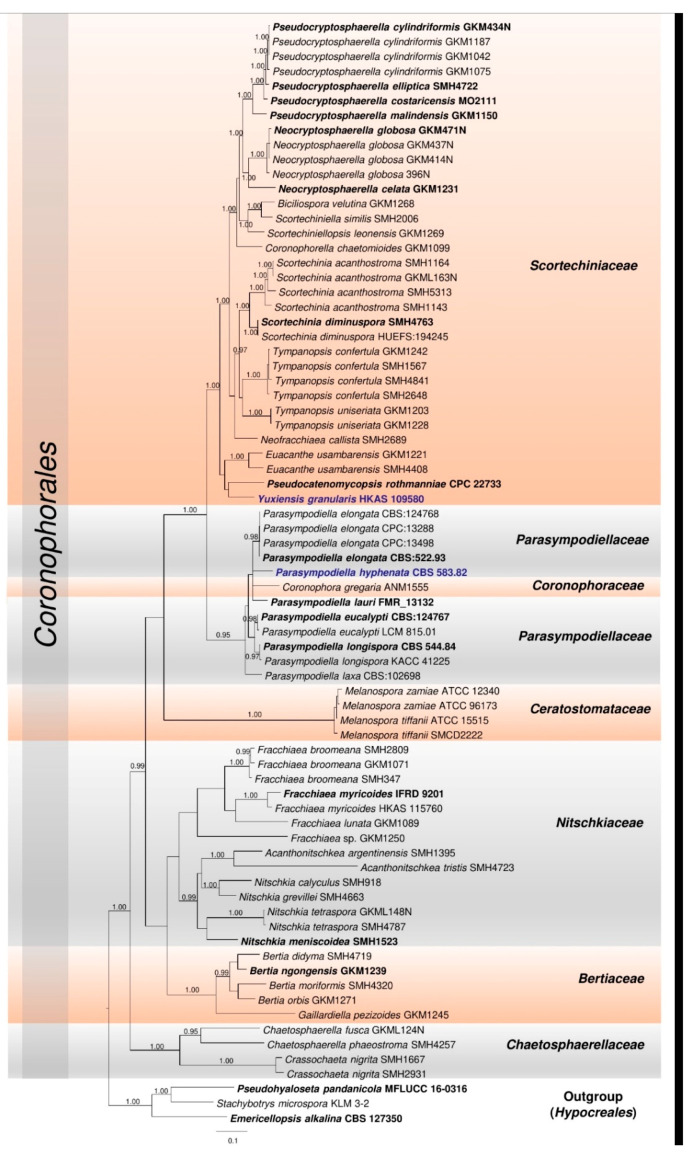
Phylogram inferred from the Bayesian analysis of LSU–ITS–*tef1*–*rpb2* matrix for *Coronophorales*. The tree is rooted with *Emericellopsis alkalina* (CBS 127350), *Pseudohyaloseta pandanicola* (MFLUCC 16-0316), and *Stachybotrys microspora* (KLM 3-2). BYPP (≥0.95 PP) supports are given above or below the branches. Type strains are in bold while novelty and the recombined taxon are in blue.

The families *Bertiaceae*, *Ceratostomataceae*, *Chaetosphaerellaceae*, *Coronophoraceae*, *Nitschkiaceae*, *Parasympodiellaceae* and *Scortechiniaceae* grouped together, each forming a monophyletic clade in the ML tree (Figure 1). The tree topology resulting from the BYPP analysis mainly differed from the ML one with regard to the placement of *Coronophora gregaria* (ANM1555) (*Coronophoraceae*), which clustered within *Parasympodiellaceae* (Figure A1).

*Parasympodiellaceae* formed a sister clade with *Scortechiniaceae* with statistical support of 96% ML, 1.00 BYPP. It comprised ten strains of *Parasympodiella* and a strain of ‘*Arthrocristula hyphenata*’ (CBS 583.82), which clustered together with 98% ML and 0.95 BYPP statistical support (Figure 1).

Our strain HKAS 109580 formed a distinct lineage within *Scortechiniaceae*, placed sister to *Pseudocatenomycopsis rothmanniae* (CPC 22733) with low statistical support (Figure 1).

### 3.2. Taxonomy

In this section, the amended descriptions and notes for *Coronophorales*, *Parasympodiellaceae,* and *Parasympodiella* are given. Furthermore, descriptions, notes, and illustrations are given for the following taxa: *Parasympodiella hyphenata* comb. nov. and *Yuxiensis granularis* gen. et sp. nov.

#### 3.2.1. *Coronophorales* Nannf., Nova Acta R. Soc. Scient. Upsal., Ser. 4 8(no. 2): 54 (1932) Amend

Index Fungorum number: IF 501516; Facesoffungi number: FoF 06517

= *Parasympodiellales* Hern.-Restr., Gené, R.F. Castañeda & Crous 2017

*Saprobic* on leaves, wood, or associated with sclerotia. Sexual morph: see Mugambi and Huhndorf [2], Hyde et al. [8], Nannfeldt [10]. Asexual morph: Hyphomycetous. *Conidiophores* simple or branched, septate, straight to flexuous, brown, smooth or comprising rough swellings along the whole length in some genera or geniculations in others. *Conidiogenous cells* apical, lateral or intercalary, usually hyaline, often sympodial, blastic or thallic. *Conidia* hyaline or brown, aseptate to septate, globose, oval, elliptical, ellipsoidal, pyriform, cylindrical, oblong or spindle-shaped, smooth or verrucose, solitary or produced in branched or unbranched chains. Synasexual morph: stylaspergillus-like (see Hernández-Restrepo et al. [13], Cheewangkoon et al. [16]), or arthrocristula-like: *Conidiophores* branched, brown, smooth. *Conidiogenous cells* apical, lateral, sympodial, thallic. *Conidia* pale brown or brown, aseptate to septate, cylindrical to oblong, produced in unbranched chains.

Type family: *Coronophoraceae* Höhn.

Notes: The phylogenetic analyses based on the combined LSU–ITS–*tef1*–*rpb2* sequence data, conducted in the present study, supports the inclusion of *Parasympodiellales* in *Coronophorales*, as *Parasympodiellaceae* is sister to *Scortechiniaceae* with 96% ML and 1.00 BYPP statistical support within the order (Figure 1). The description for the asexual morph of taxa in *Coronophorales* is therefore emended to include the morphological characteristics of *Parasympodiellaceae*. 

#### 3.2.2. *Parasympodiellaceae* Hern.-Restr., Gené, Guarro & Crous, in Hernández-Restrepo, Gené, Castañeda-Ruiz, Mena-Portales, Crous & Guarro, Stud. Mycol. 86: 87 (2017) Amend

Index Fungorum number: IF 820298; Facesoffungi number: FoF 06518

*Saprobic* on leaves and twigs or associated with sclerotia. Sexual morph: Undetermined. Asexual morph: Hyphomycetous. *Conidiophores* micro- to macronematous, brown, usually unbranched, septate. *Conidiogenous cells* thallic, terminal or intercalary, sympodial. *Conidia* thallic-arthric, aseptate or septate, cylindrical, hyaline, often in chains, with schizolytic secession. Synasexual morph: stylaspergillus-like (see Hernández-Restrepo et al. [13], Cheewangkoon et al. [16]) or arthrocristula-like: *Conidiophores* micro- to macronematous, brown, branched. *Conidiogenous cells* thallic, terminal, lateral, sympodial. *Conidia* thallic-arthric, aseptate or septate, cylindrical to oblong, pale brown or brown, generally in chains, with rhexolytic secession.

Type genus: *Parasympodiella* Ponnappa

Notes: *Parasympodiellaceae* is now included in *Coronophorales*, which at present comprises seven families (this study). This family, in turn, accommodates the single genus *Parasympodiella*.

#### 3.2.3. *Parasympodiella* Ponnappa Trans. Br. Mycol. Soc. 64(2): 344 (1975) Amend

Index Fungorum number: IF 9226; Facesoffungi number: FoF 05188

= *Arthrocristula* Sigler, M.T. Dunn & J.W. Carmich., Mycotaxon 15: 409 (1982)

*Saprobic* on leaves and twigs or associated with sclerotia. Sexual morph: Undetermined. Asexual morph: Hyphomycetous. *Conidiophores* micro- to macronematous, brown, generally unbranched, septate, straight to geniculate. *Conidiogenous cells* thallic, terminal or intercalary, indeterminate, sympodial, unbranched. *Conidia* thallic-arthric, produced in unbranched chains, cylindrical, aseptate or septate, hyaline, seceding schizolytically, often with a septal plug. Synasexual morph: stylaspergillus-like (see Hernández-Restrepo et al. [13], Cheewangkoon et al. [16]) or arthrocristula-like: *Conidiophores* micro- to macronematous, brown, branched. *Conidiogenous cells* thallic, terminal, lateral, indeterminate, sympodial, unbranched. *Conidia* thallic-arthric, produced in unbranched chains, aseptate or septate, pale brown or brown, cylindrical to oblong, with rhexolytic secession, with frills of remnant cells at each end.

Type species: *Parasympodiella laxa* (Subram. & Vittal) Ponnappa

Notes: With the exact taxonomic placement being uncertain, the hyphomycetous taxon *Arthrocristula* has so far been maintained in *Ascomycota* genera *incertae sedis* [18,38]. In the present study, the type strain of *A. hyphenata* (CBS 583.82) was found to cluster within *Parasympodiellaceae*, indicating a close phylogenetic affinity to *Parasympodiella* (Figure 1). Morphologically, the conidiophores of *Parasympodiella* are unbranched or sparingly branched, thick-walled, and brown, becoming paler towards the conidiogenous regions. They are often geniculate, with terminal or intercalary sympodial conidiogenous cells, which are mostly thallic. The secession of conidia occurs schizolytically [13,14,16]. The conidiophores of *Arthrocristula* are, however, well-branched with no conspicuous geniculation and are initially hyaline, becoming thick-walled and brown upon maturity. The conidiogenous cells secede rhexolytically into arthroconidia, leaving the mature conidia with remnants of the separating cells at each end with small frills. The terminal cells of the conidiogenous hyphae remain as empty cells [17]. Considering these morphological differences, the two genera can be considered as distinct. However, given that *Parasympodiella* has a stylaspergillus-like synasexual morph, the fact that *Arthrocristula* also is another synasexual morph of *Parasympodiella* cannot be ruled out. Fungi have been reported to have two or more morphologically distinct asexual morphs [39,40,41]. Moreover, phylogeny supports the inclusion of *Arthrocristula* in *Parasympodiella* (Figure 1). Therefore, the former is synonymized under *Parasympodiella* in the present study.

#### 3.2.4. *Parasympodiella hyphenata* (Sigler, M.T. Dunn & J.W. Carmich.) Bundhun & K.D. Hyde, comb. nov.

**Figure 2:**
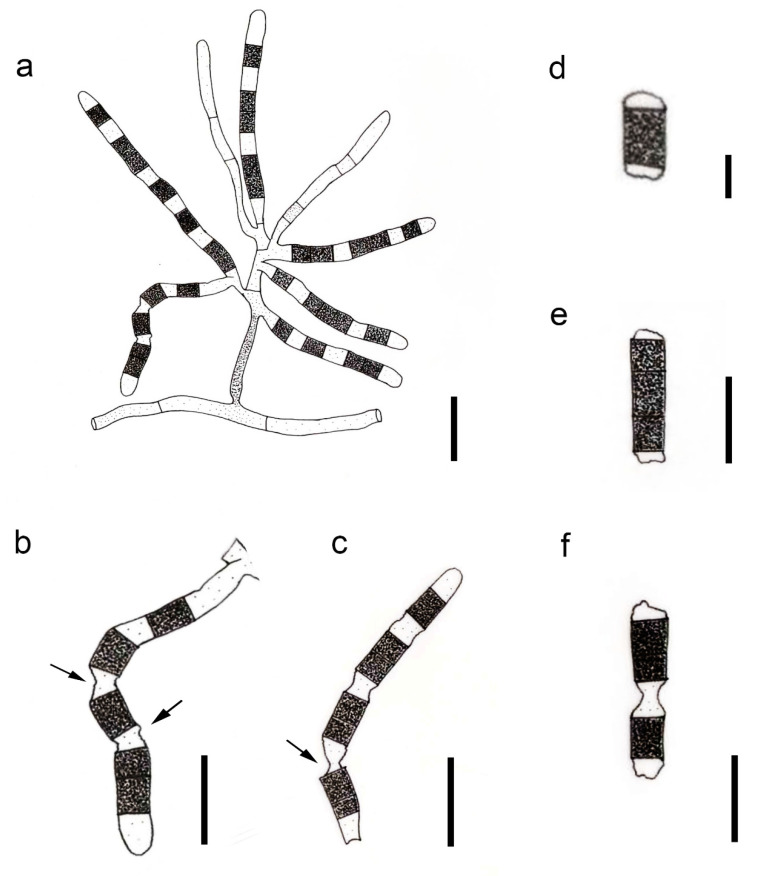
*Parasympodiella hyphenata* redrawn from Sigler et al. [17] and Seifert et al. [42]. (**a**) Conidiogenous hyphae originating from a conidiophore. (**b**,**c**) Conidiogenous hyphae, with rhexolytically dehiscing intervening cells (shown by arrows) and darker pigmented area denoting conidia. (**d**–**f**) Arthroconidia with remnants of cell walls attached at both ends. Scale bars: (**a**–**c**) = 30 µm, (**d**) = 5 µm, (**e**,**f**) = 10 µm (scale bars adapted based on original description in Sigler et al. [17]).

Index Fungorum number: IF 558677; Facesoffungi number: FoF 10183, Figure 2

≡ *Arthrocristula hyphenata* Sigler, M.T. Dunn & J.W. Carmich., Mycotaxon 15: 409 (1982)

Associated with sclerotia of *Sclerotinia minor* in soil. Sexual morph: Undetermined. Asexual morph: Hyphomycetous. *Mycelium in vitro* comprises narrow, septate vegetative hyphae (2–3 µm diam.), hyaline when immature and brown at maturity. *Conidiophores* originating from vegetative hyphae, undifferentiated, initially hyaline, turning brown and thick-walled when mature, branched, narrow at first, widening and extending apically and laterally after that to give rise to fertile conidiogenous hyphae. *Conidiogenous cells* (80–100 × 4–5.5 µm), delimited by a basal septum, developing successively, generally unbranched, indeterminate, initially non-septate, hyaline, becoming randomly septate and pigmented in alternate cells on maturity, undergoing rhexolytic secession to give rise to arthroconidia. *Conidia* (5–12 × 6–7 µm), thallic-arthric, brown, aseptate to septate, cylindrical to oblong, formed in a chain with hyaline separating cells, with fragments of intervening cell walls remaining attached to both ends of conidium after secession, terminal cells of the chains remain empty. *Chlamydospores in vitro* (12–16 × 8–13 µm), thick-walled, brown, terminal and intercalary (adapted from Sigler et al. [17]). 

Notes: The type and single taxon of *Arthrocristula*, *A. hyphenata* is combined under *Parasympodiella* following the synonymy of *Arthrocristula* under *Parasympodiella* in the present study. *Parasympodiella hyphenata* comb. nov. has been reported from the sclerotium of *Sclerotinia minor* buried in the soil [17]. It is mainly characterized by branched conidiophores and alternatively pigmented conidiogenous cells, which produce conidia by seceding rhexolytically [17].

#### 3.2.5. *Yuxiensis* Bundhun & K.D. Hyde, gen. nov.

Index Fungorum number: IF 558675; Facesoffungi number: FoF 10184

Etymology–Referring to the city Yuxi in China.

*Saprobic* on dead wood. Sexual morph: *Ascomata* immersed to erumpent, appearing superficial on worn off substrate, astromatic to stromatic, aggregated, black, non-ostiolate, semi-globose when fresh, collapsing when dry, without hair or bristles, subiculum inconspicuous, coriaceous. *Ascomatal wall* comprising 2–3 types of layers; outermost layer heavily pigmented, composed of thick-walled, very dark brown cells, inner layer comprising thick-walled cells of *textura globulosa* to *textura angularis*, innermost layer composed of flattened, thin-walled cells of *textura prismatica* toward the locule; *Munk pores* visible, few per cell. *Hamathecium* composed of large, subcylindrical quellkörper, attached to the roof of the centrum and extending until base of the ascoma; paraphyses indistinct. *Asci* 8-spored, unitunicate, clavate, long-pedicellate, rounded at apex, lacking an apical ring, thin-walled, evanescent. *Ascospores* irregularly arranged, cylindrical to allantoid, hyaline, aseptate, with granular contents, lacking mucilaginous sheath or appendage. Asexual morph: Undetermined.

Type: *Yuxiensis granularis* Bundhun, Wanas. & K.D. Hyde

Notes: *Yuxiensis* is introduced in *Scortechiniaceae* as a new quellkörper-bearing taxon distinct from all other genera in the family, based on LSU, ITS, *tef1* and *rpb2* sequence data. The new genus has a close phylogenetic affinity to *Pseudocatenomycopsis* even though this relationship is not statistically significant (Figure 1). This low support may possibly be accounted for by insufficient taxon sampling. Nevertheless, the new taxon being introduced in the present study constantly clusters with *Pseudocatenomycopsis* in all phylogenies (single, not shown; and concatenated, Figure 1). The asexual morph for *Yuxiensis* could not be obtained in the present study. It thus cannot be morphologically compared with *Pseudocatenomycopsis*, which has been described in its asexual morph only. *Pseudocatenomycopsis* has been introduced from Zambia as a saprobe on the stem of *Rothmannia engleriana* (*Rubiaceae*) [11]. The new genus is also phylogenetically close to *Euacanthe* (Figure 1). It morphologically differs from *Euacanthe* in terms of ascomatal position and surface as well as ascospore ornamentation [12,43]. 

#### 3.2.6. *Yuxiensis granularis* Bundhun, Wanas. & K.D. Hyde, sp. nov.

**Figure 3:**
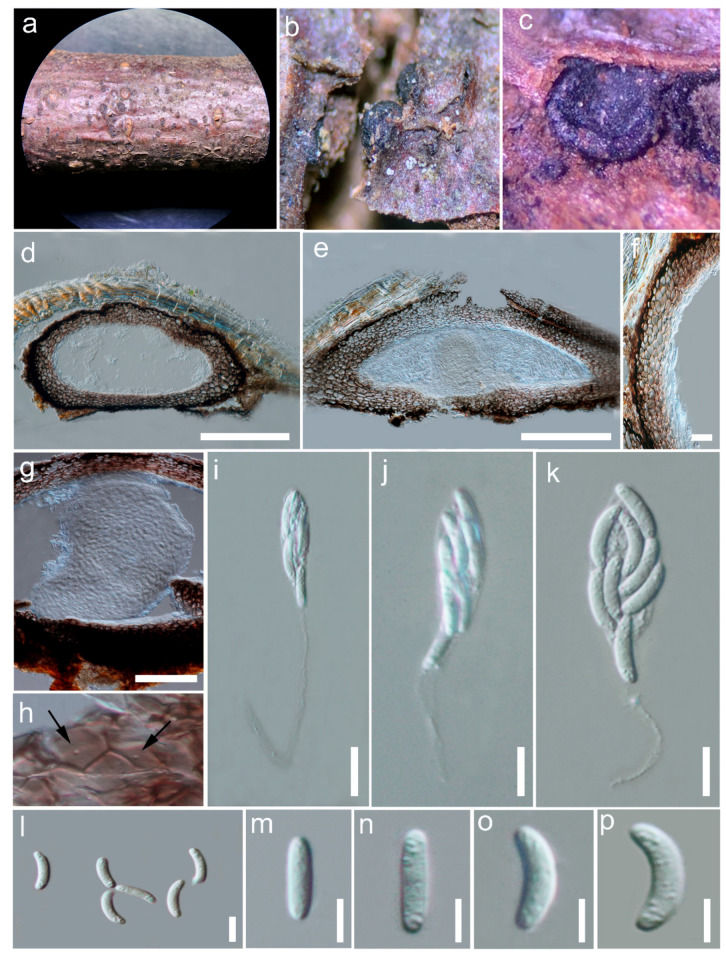
*Yuxiensis granularis* (HKAS 109580, holotype). (**a**) Appearance of ascomata on twig. (**b**) Close-up of ascomata. (**c**) Collabent ascoma. (**d**,**e**) Longitudinal sections of ascoma. (**f**) Peridium. (**g**) Quellkörper. (**h**) Munk pores (arrows). (**i**–**k**) Asci. (**l**–**p**) Ascospores. Scale bars: (**d**,**e**) = 200 µm, (**f**) = 30 µm, (**g**) = 100 µm, (**i**–**k**) = 10 µm, (**l**–**p**) = 5 µm.

Index Fungorum number: IF 558676; Facesoffungi number: FoF 10185, Figure 3

Etymology—The specific epithet refers to the granular contents of the ascospores.

Holotype–HKAS 109580

*Saprobic* on dead twigs of deciduous hosts in terrestrial habitats. Sexual morph: *Ascomata* 250–400 μm high, 550–700 μm diam. ($\bar{x}$ = 309 × 642 μm, n = 5), astromatic to stromatic, immersed to erumpent, appearing superficial after substrate has worn away, aggregated, black, non-ostiolate, semi-globose when fresh, collapsing upon drying, without hair or bristles, with inconspicuous subiculum, coriaceous. *Ascomatal wall* made up of 2–3 layers, almost equally thickened, 40–70 μm wide at the apex and base, 50–70 μm wide at the sides; heavily pigmented at outermost layer, composed of thick-walled, very dark brown cells, with inner layer comprising thick-walled cells (25–45 μm) of *textura globulosa* to *textura angularis*, innermost layer composed of flattened, thin-walled cells of *textura prismatica* toward the locule; *Munk pores* visible, few per cell. *Hamathecium* made up of subcylindrical quellkörper, 290 μm long and 220 μm wide. *Asci* 40–80 × 5–10 μm ($\bar{x}$ = 55.8 × 8.4 μm, n = 10), spore bearing part 15–30 μm, pedicel 15–50 μm, 8-spored, unitunicate, clavate, long-pedicellate, rounded at apex, lacking an apical ring, thin-walled, evanescent. *Ascospores* 8–15 × 2–3 μm ($\bar{x}$ = 11.7 × 2.3 μm, n = 35), irregularly arranged, cylindrical to allantoid, hyaline, unicellular, aseptate, with granular contents, lacking mucilaginous sheath or appendage. Asexual morph: Undetermined.

Material examined: CHINA, Yunnan, Yuxi, Yi and Dai Autonomous County, Yuanjiang Hani, 23.74074 N, 102.17735 E, on woody litter of an undetermined deciduous host, 24 May 2019, 1345 msl, D.N. Wanasinghe, DW0636-19 (HKAS 109580, holotype). 

Notes: In the multi-gene phylogeny, *Yuxiensis granularis* is more closely related to *Pseudocatenomycopsis rothmanniae*, followed by *Euacanthe usambarensis* (=*Euacanthe foveolata* [12]) (Figure 1). The LSU sequence of *Yuxiensis granularis* is 95% similar to *Pseudocatenomycopsis rothmanniae* (GenBank KF777237; similarity = 869/910(95%), Gaps = 3/910(0%)). The ITS sequence of *Yuxiensis granularis* is 85% similar to *Pseudocatenomycopsis rothmanniae* (GenBank KF777185; similarity = 470/552(85%), Gaps = 10/552(1%)). *Pseudocatenomycopsis rothmanniae* has only LSU and ITS sequence data deposited in GenBank, and hence the protein-coding genes, *tef1* and *rpb2* could not be compared. Morphological comparison between the two taxa is currently unfeasible since the single species of *Pseudocatenomycopsis*, *P. rothmanniae*, has been introduced in its asexual morph [11], while the asexual morph for *Yuxiensis granularis* could not be obtained. 

*Yuxiensis granularis* resembles *Euacanthe usambarensis* in having ascomata which become collabent on drying and 8-spored, clavate asci [2,12,43]. However, the new species has immersed to erumpent, glabrous ascomata that are not surrounded by any conspicuous subiculum or tomentum, whereas *Euacanthe usambarensis* comprises superficial ascomata with a setose surface, sitting on a dense subiculum [12,43]. The asci of *Yuxiensis granularis* are long-pedicellate whereas those of *Euacanthe usambarensis* have short pedicels [12,43]. Furthermore, while the ascospore contents of *Yuxiensis granularis* are granular, those of *Euacanthe usambarensis* generally have two guttules [12,43]. There are also more than 2.5% nucleotide differences in LSU out of 925 base pairs among the strains of *Yuxiensis granularis* (HKAS 109580) and *Euacanthe usambarensis* (GKM1221 and SMH4408), while more than 2.5% nucleotide differences in *tef1* and *rpb2* out of 731 and 1178 base pairs respectively between the strains of *Yuxiensis granularis* (HKAS 109580) and *Euacanthe usambarensis* (GKM1221).

## 4. Discussion

*Sordariomycetes* is a frequently-studied class, with several taxa having been recently introduced or revised [4,5,8,12,44]. The present study corroborates this fact, as supported, firstly, by establishing a new saprobic genus, *Yuxiensis,* in *Scortechiniaceae* based on a dual taxonomic approach. In addition to phylogeny, the familial placement of the new genus within *Scortechiniaceae* is morphologically confirmed by the presence of the quellkörper in its centrum. Within *Scortechiniaceae*, *Yuxiensis* shares many overlapping characters with the other genera. For instance, like almost all the other genera in the family with a known sexual morph, *Yuxiensis* comprises ascomata which collapse upon drying, presence of munk pores in the ascomatal wall and inconspicuous paraphyses [4,8]. It also has 8-spored asci, similar to *Biciliospora*, *Coronophorella*, *Euacanthe*, *Scortechinia*, and *Tympanopsis* and long-pedicellate asci like *Biciliospora*, *Neofracchiaea*, *Scortechinia*, *Scortechiniella,* and *Scortechiniellopsis* [2,4,8]. It is equally similar to most taxa of *Neocryptosphaerella* and *Pseudocryptosphaerella* in that its ascomata are immersed to erumpent, appearing superficial when the substrate has worn away [2,12]. The new genus however, demarcates itself from the other genera in the family by several ways. Its ascomata are not seated on or surrounded by a well-developed, conspicuous subiculum unlike many taxa of *Biciliospora*, *Coronophorella*, *Euacanthe*, *Neofracchiaea*, *Scortechinia*, *Scortechiniella*, *Scortechiniellopsis,* or *Tympanopsis* [2,4,8]. The ascomata of *Yuxiensis* are also devoid of a tomentose or setose surface as compared to *Euacanthe*, *Neofracchiaea,* or some taxa of *Neocryptosphaerella* and *Pseudocryptosphaerella* [2,8]. Moreover, the ascospores of *Yuxiensis* do not have conspicuous guttules unlike those of *Euacanthe*, *Neocryptosphaerella,* and *Pseudocryptosphaerella* taxa and they lack appendage-like wall extensions on both ends, contrary to *Biciliospora* and *Scortechiniella* [2,45]. Since *Yuxiensis* is phylogenetically closely related to *Pseudocatenomycopsis* and *Euacanthe* (Figure 1), more details about their morpho-molecular comparisons have been given in the result parts 3.2.5 and 3.2.6 above. Based on all these morphological as well as phylogenetic differences, *Yuxiensis* is herein introduced as a new genus. 

The inclusion of *Parasympodiellales* in *Coronophorales* in the present study also points toward the continuous amendment in the classification of *Sordariomycetes.* Herein, while phylogeny supports the addition of *Parasympodiellaceae* to *Coronophorales*, this inclusion is equally supported by the fact that taxa of *Parasympodiellaceae* have similar morphological characters with several taxa in other families of *Coronophorales* (*Ceratostomataceae*, *Chaetosphaerellaceae*, *Scortechiniaceae* ) in terms of unbranched or branched, generally brown and often septate conidiophores or conidia produced in chains [4,8,11]. *Parasympodiellaceae* distinguishes itself from the other families mainly by its sympodial and unbranched conidiogenous cells which undergo schizolytic or rhexolytic secession to form arthroconidia. Furthermore, the *incertae sedis* taxon *Arthrocristula* is synonymized under *Parasympodiella*, with *Arthrocristula hyphenata* recombined to *Parasympodiella hyphenata* and representing another synasexual morph of *Parasympodiella.* This arthrocristula-like synasexual morph of *Parasympodiella* is typically characterized by branched conidiophores and conidiogenous cells which secede rhexolytically to give rise to arthroconidia. It is also different from the stylaspergillus-like synasexual morph of *Parasympodiella* which is generally characterized by pale brown, phialidic conidiogenous cells originating from terminal or intercalary vesicle-like cells and filiform conidia which are produced in slimy masses [13].

An additional collection of *Fracchiaea myricoides* (HKAS 115760) was also made in the present study, and sequence data for the same have been used in the phylogenetic analyses and deposited in GenBank (Table 1). The latter species was initially introduced as *Coronophora myricoides* based on LSU and ITS sequence data and the differences mentioned between this taxon and the type species of *Coronophora*, *C. gregaria* was mainly based on the shapes of the ascomata and ascospores [46]. Huang et al. [12] recently synonymized this species to *Fracchiaea myricoides*; our collection supports this synonymy and the inclusion of ‘*Coronophora myricoides*’ in *Fracchiaea* (*Nitschkiaceae*) (Figure 1). 

Despite the advancement towards a natural classification of *Sordariomycetes*, uncertainties and confusions still prevail, as we note in the case of *Parasympodiella longispora* (CBS 544.84 and KACC 41225) (in *Parasympodiellaceae* clade, Figure 1). The latter is currently known as ‘*Bahusakala longispora*’ in Index Fungorum and MycoBank, with *Parasympodiella longispora* as an (obligate) synonym. The type strain of ‘*Bahusakala longispora*’, CBS 544.84, sequenced by Vu et al. [19], clusters in the *Parasympodiellaceae* clade (Figure 1) with good statistical support. *Bahusakala longispora* was introduced by Tokumasu and Tubaki [47] as a taxon with conidiophores that are sympodial, rarely branched, erect in the lower part and become geniculate (zig-zag, as mentioned in the original description) in the upper part. Furthermore, the conidiogenous cells are hyaline, originating at regular intervals, while the arthroconidia, subhyaline to pale yellow. Chlamydospores are produced in vegetative hyphae. However, the species was later synonymized to *Parasympodiella longispora* since its morphology (based on its type) matched the description of *Parasympodiella* more accurately [48]. *Bahusakala* taxa have been reported to have conidiophores that branch at irregular intervals to produce brown conidiogenous hyphae at the terminal and intercalary positions. The conidia are usually brown to dark brown and originate from random disarticulation of the main conidiophore axes and conidiogenous hyphae [42,48,49,50]. Based on the description of its type (and placement in the present phylogenetic tree), the species is better accommodated in *Parasympodiella* than *Bahusakala*. We may as well adopt a broader taxonomic perspective and decide that both *Parasympodiella* and *Bahusakala* are congeneric since, despite their morphological differences, the two genera are also characterized by similar features. Both are hyphomycetes with erect and septate conidiophores and produce arthroconidia which secede schizolytically [42,50]. However, no molecular data for the type species, *B. olivaceonigra* is yet available to enable any definite phylogenetic placement and eventually to confirm a taxonomic conclusion for *Bahusakala*. 

We also take note that the GenBank accession numbers of the sequence data for *Neocryptosphaerella globosa* (GKM471N) that we use in our phylogeny have been assigned under different strain numbers, namely, LSU (GenBank FJ968977: strain GKM469N), *tef1* (GenBank FJ969036: strain GKM471N), and *rpb2* (GenBank FJ968935: strain GKM469N). In the original manuscript [2], these accession numbers are under the strain number *Neocryptosphaerella globosa* GKM471N. Therefore, we followed the original paper. 

This remarkable finding of a new genus in a rarely collected order indicates how little we know of the fungal diversity of Yunnan and the broader region, including areas such as Thailand and Laos [51], with recent studies showing large numbers of novel taxa being discovered [52,53]. Further studies in other countries and habitats across this region will surely result in the discovery of numerous other taxa in *Parasympodiella*, *Yuxiensis,* and other poorly known taxa of *Coronophorales* [54].

## Figures and Tables

**Table 1:** Taxa used in this study and their corresponding GenBank accession numbers. Generated sequence data for the new taxon are in bold.

Taxa	Strains	GenBank Accession Numbers			
		LSU	ITS	*tef* *1*	*rpb* *2*
*Acanthonitschkea argentinensis*	SMH1395	AY695259	-	FJ969042	FJ968943
*Acanthonitschkea tristis*	SMH4723	FJ968949	-	FJ969043	-
*Bertia didyma*	SMH4719	FJ968958	-	-	-
*Bertia ngongensis*	GKM1239 *	FJ968954	-	-	-
*Bertia moriformis*	SMH4320	AY695260	-	-	AY780151
*Bertia orbis*	GKM1271	FJ968955	-	FJ969009	-
*Biciliospora velutina*	GKM1268	FJ968964	-	FJ969018	FJ968932
*Chaetosphaerella fusca*	GKML124N	FJ968967	-	FJ969002	-
*Chaetosphaerella phaeostroma*	SMH4257	AY695264	-	FJ969004	FJ968940
*Coronophora gregaria*	ANM1555	-	-	FJ969007	FJ968938
*Coronophorella chaetomioides*	GKM1099	FJ968969	-	FJ969034	FJ968922
*Crassochaeta nigrita*	SMH1667	AY695265	-	-	-
	SMH2931	AY695266	-	-	-
*Emericellopsis alkalina*	CBS 127350 *	MH875970	MH864534	KC998993	KC999029
*Euacanthe usambarensis*	GKM1221	FJ968978	-	FJ969026	FJ968927
	SMH4408	AY695267	-	-	-
*Fracchiaea broomeana*	SMH347	FJ968979	-	FJ969041	FJ968947
	SMH2809	AY695268	-	FJ969039	FJ968942
	GKM1071	-	-	FJ969040	FJ968919
*Fracchiaea myricoides*	IFRD 9201 *	KX856174	KX856173	-	-
	HKAS 115760	MZ713199	MZ713184	MZ712579	MZ712580
*Fracchiaea lunata*	GKM1089	-	-	-	FJ968921
*Fracchiaea* sp.	GKM1250	-	-	FJ969005	-
*Gaillardiella pezizoides*	GKM1245	FJ968981	-	FJ969006	-
*Melanospora tiffanii*	ATCC 15515	AY015630	-	-	AY015637
	SMCD2222	FJ748915	FJ748921	-	-
*Melanospora zamiae*	ATCC 12340	AY046579	-	-	AY046580
	ATCC 96173	AY057906	-	-	-
*Neocryptosphaerella celata*	GKM1231 *	FJ968975	-	FJ969035	FJ968929
*Neocryptosphaerella globosa*	GKM471N *	FJ968977	-	FJ969036	FJ968935
	GKM437N	-	-	FJ969038	-
	GKM414N	-	-	FJ969037	-
	396N	FJ968976	-	-	-
*Neofracchiaea callista*	SMH2689	AY695269	-	FJ969020	FJ968941
*Nitschkia calyculus*	SMH918	FJ968983	-	-	-
*Nitschkia grevillei*	SMH4663	AY346294	-	-	-
*Nitschkia meniscoidea*	SMH1523 *	AY695270	-	-	-
*Nitschkia tetraspora*	GKML148N	FJ968987	-	FJ969011	FJ968936
	SMH4787	FJ968984	-	FJ969010	-
*Parasympodiella elongata*	CBS:522.93 *	GQ303314	GQ303283	-	-
	CBS:124768	GQ303311	GQ303280	-	-
	CPC:13288	GQ303312	GQ303281	-	-
	CPC:13498	GQ303313	GQ303282	-	-
*Parasympodiella eucalypti*	CBS:124767 *	GQ303315	GQ303284	-	-
	LCM 815.01	-	MF495381	-	-
*Parasympodiella hyphenata*	CBS 583.82 *	MH873274	MH861530	-	-
*Parasympodiella lauri*	FMR_13132 *	KY853518	KY853457	-	-
*Parasympodiella laxa*	CBS 102698	GQ303316	GQ303285	-	-
*Parasympodiella longispora*	CBS 544.84 *	MH873476	MH861778	-	-
	KACC 41225	-	GQ272636	-	-
*Pseudocatenomycopsis rothmanniae*	CPC 22733 *	KF777237	KF777185	-	-
*Pseudocryptosphaerella costaricensis*	MO2111 *	FJ968971	-	FJ969028	-
*Pseudocryptosphaerella cylindriformis*	GKM434N *	FJ968972	-	FJ969031	FJ968934
	GKM1187	GQ217531	-	FJ969033	FJ968925
	GKM1042	FJ968973	-	FJ969032	FJ968918
	GKM1075	-	-	FJ969030	FJ968920
*Pseudocryptosphaerella elliptica*	SMH4722 *	FJ968974	-	FJ969029	FJ968944
*Pseudocryptosphaerella malindensis*	GKM1150 *	FJ968970	-	FJ969027	FJ968923
*Pseudohyaloseta pandanicola*	MFLUCC 16-0316 *	MH376737	MH388363	MH388398	MH412733
*Scortechinia acanthostroma*	SMH1164	FJ968989	-	FJ969014	FJ968924
	SMH1143	FJ968988	-	FJ969012	FJ968948
	GKML163N	FJ968991	-	FJ969015	-
	SMH5313	FJ968990	-	FJ969013	-
*Scortechinia diminuspora*	SMH4763 *	FJ968992	-	-	-
	HUEFS:194245	KT003703	-	-	-
*Scortechiniella similis*	SMH2006	FJ968994	-	FJ969019	FJ968945
*Scortechiniellopsis leonensis*	GKM1269	FJ968993	-	FJ969021	FJ968933
*Stachybotrys microspora*	KLM 3-2	KU760387	KU760377	KU760392	KU760397
*Tympanopsis confertula*	GKM1242	FJ968997	-	FJ969023	FJ968930
	SMH1567	FJ969001	-	-	FJ968939
	SMH4841	FJ968998	-	FJ969024	FJ968946
	SMH2648	AY695272	-	-	-
*Tympanopsis uniseriata*	GKM1203	FJ968999	-	FJ969016	FJ968926
	GKM1228	FJ969000	-	FJ969017	-
***Yuxiensis granularis***	HKAS 109580 *	**MZ713198**	**MZ713183**	**MZ712577**	**MZ712578**

Type strains are indicated in ‘*’.

## Data Availability

The nucleotide sequences generated in the present study are deposited in GenBank (Table 1). The final alignment and phylogenetic tree have been submitted to TreeBASE (submission ID: 28713, http://www.treebase.org/, accessed on 30 August 2021). Specimen has been deposited in the herbarium of Cryptogams Kunming Institute of Botany Academia Sinica (HKAS).
